# Development of the probability of return of spontaneous circulation in intervals without chest compressions during out-of-hospital cardiac arrest: an observational study

**DOI:** 10.1186/1741-7015-7-6

**Published:** 2009-02-06

**Authors:** Kenneth Gundersen, Jan Terje Kvaløy, Jo Kramer-Johansen, Petter Andreas Steen, Trygve Eftestøl

**Affiliations:** 1 Department of Electrical and Computing Engineering, University of Stavanger, Stavanger, Norway; 2 Department of Mathematics and Natural Sciences, University of Stavanger, Stavanger, Norway; 3 Institute for Experimental Medical Research, Ulleval University Hospital, Oslo, Norway; 4 Faculty Division UUS, University of Oslo, Oslo, Norway; 5 Division of Prehospital Emergency Medicine, Ulleval University Hospital, Oslo, Norway

## Abstract

**Background:**

One of the factors that limits survival from out-of-hospital cardiac arrest is the interruption of chest compressions. During ventricular fibrillation and tachycardia the electrocardiogram reflects the probability of return of spontaneous circulation associated with defibrillation. We have used this in the current study to quantify in detail the effects of interrupting chest compressions.

**Methods:**

From an electrocardiogram database we identified all intervals without chest compressions that followed an interval with compressions, and where the patients had ventricular fibrillation or tachycardia. By calculating the mean-slope (a predictor of the return of spontaneous circulation) of the electrocardiogram for each 2-second window, and using a linear mixed-effects statistical model, we quantified the decline of mean-slope with time. Further, a mapping from mean-slope to probability of return of spontaneous circulation was obtained from a second dataset and using this we were able to estimate the expected development of the probability of return of spontaneous circulation for cases at different levels.

**Results:**

From 911 intervals without chest compressions, 5138 analysis windows were identified. The results show that cases with the probability of return of spontaneous circulation values 0.35, 0.1 and 0.05, 3 seconds into an interval in the mean will have probability of return of spontaneous circulation values 0.26 (0.24–0.29), 0.077 (0.070–0.085) and 0.040(0.036–0.045), respectively, 27 seconds into the interval (95% confidence intervals in parenthesis).

**Conclusion:**

During pre-shock pauses in chest compressions mean probability of return of spontaneous circulation decreases in a steady manner for cases at all initial levels. Regardless of initial level there is a relative decrease in the probability of return of spontaneous circulation of about 23% from 3 to 27 seconds into such a pause.

## Background

Recent evidence indicates that cardiopulmonary resuscitation (CPR) during both in- and out-of-hospital cardiac arrest is characterised by frequent and long interruptions in chest compressions [1,2]. This reduces vital organ perfusion [3], and in animal experiments, increased length of chest compression pause before shock correlates with reduced rates of return of spontaneous circulation (ROSC) and survival [4-6]. Edelson et al. [7] reported that successful defibrillation, defined as removal of ventricular fibrillation (VF) for at least 5 seconds, was associated with shorter pre-shock pauses in man. Eilevstjønn et al. [8] reported a similar association for shocks with ROSC outcome but only reported the median length of pre-shock pauses for ROSC and no-ROSC shocks. Identifying in detail how pausing in chest compressions affects the vitality of the myocardium, and thereby the probability of ROSC (*P*_ROSC_) after defibrillation, is important because it affects treatment priorities before a defibrillation, a vital stage of the resuscitation effort.

During VF and ventricular tachycardia (VT) one can calculate ROSC-predictors from the electrocardiogram (ECG) reflecting the *P*_ROSC _associated with defibrillation [9-12]. In general terms, we can say that ROSC-predictors reflect the coarseness of the ECG or the vitality of the myocardium. ROSC-predictors have been affected positively by compression sequences both in animals [13] and man [14] and negatively by periods with no chest compressions both in animals [15] and man [16]. Unfortunately, we have now realised that the statistical analysis performed in the last article [16] was flawed. This is explained in Appendix 1. In the current work therefore, we reinvestigate the effect of interruptions of chest compressions on *P*_ROSC _calculated from the ECG. Our hypothesis was that *P*_ROSC _decreases during such interruptions and that the size of this effect may depend on the absolute value of *P*_ROSC_. We use a ROSC-predictor that represents the most accurate, currently available estimate of *P*_ROSC _[11], and a statistical methodology that properly handles short time variations of the *P*_ROSC _estimate and the fact that the *P*_ROSC _level varies from interval to interval [17]. We handle the problem not solved in Eftestol et al. [16] by applying an adequate regression to the data, representing the underlying trends in *P*_ROSC _development, and use this to derive our results. Further, we compare the results with relevant data from investigations of animal and human sudden cardiac arrest data.

## Methods

Data were collected by the respective emergency medical services in an observational prospective study of out-of-hospital cardiac arrest patients in Akershus (Norway), Stockholm (Sweden) and London (UK) in the period March 2002 to September 2004 [1,18]. The appropriate ethical boards at each site approved the study, and the need for informed consent from each patient was waived as decided by these boards in accordance with paragraph 26 of the Helsinki declaration for human medical research. The study is registered as a clinical trial at , (NCT00138996). Continuous ECG, transthoracic impedance, and chest compression depth measurements were collected using a modified Heartstart 4000 (Phillips Medical Systems, Andover, MA, USA) (Heartstart 4000SP (Laerdal Medical, Stavanger, Norway)) and patient records registered according to the Utstein template [19]. The ECG was obtained through the defibrillator's self-adhesive defibrillation pads positioned in lead II equivalent positions and the same types of electrodes were used throughout the data collection. The signal was digitally recorded with a sampling rate of 500 samples per second and 16 bits resolution. Before digital sampling the analogue ECG signal was filtered with a second order bandpass filter with passband of 0.9–50 Hz and before analysis a 48 tap lowpass digital filter with an upper passband edge of 30 Hz was applied to the digitised ECG to remove any 50 Hz power line noise. Regarding the mapping-dataset (described below) ROSC was indicated either by a clinically detected pulse or by changes in the transthoracic impedance >50 mΩ coincident with QRS complexes [1]. Compression depth measurements were used to identify the presence and absence of chest compressions. Further details about registrations and methodology have been given elsewhere [1].

### Intervals without chest compressions

We extracted ECG segments from intervals without chest compressions following an interval with compressions. Only segments with VF or VT during both intervals were included. The ECG segments were then divided into 2-second analysis windows centred at 3, 5, 7 and so on up to 27 seconds into the pause, depending on the length of the pause. The first and last 2 seconds of each interval were left out to ensure that the signals were uncorrupted by compression artefacts. We will refer to this dataset as the interval-dataset. ECG segments were manually checked for noise and noisy parts of ECG segments were censored from further analysis. The definition of noise was influence from a pacemaker (regular spikes on the ECG) or short bursts of high frequency signal that visually differ substantially from the surrounding VF or VT. The source of the latter noise form might be electrode noise or muscle artefacts.

### Outline of analysis

The ECG from the analysis windows in the interval-dataset was characterised by computing the logarithm of the mean-slope (*logslope*), one of the most accurate indicators of *P*_ROSC _[11]. Mean-slope can be viewed as a measurement of the coarseness of the ECG. High amplitude and frequency of the ECG give high mean-slope values, indicating a high *P*_ROSC_. If ecg(n) is the digitised ECG signal, *logslope *is defined as

(1) $$logslope=\ln\left(\frac{1}{N}\sum_{1}^{N}\left|ecg\left(n\right)-ecg(n-1)\right|\right)$$

*Logslope *values have the desirable property of having an approximate Gaussian distribution, and we thus expected an approximately linear decay with time in untreated VF/VT. A linear mixed-effects model was therefore fitted to *logslope *versus time. Then a mapping from *logslope *to *P*_ROSC _scale, obtained from a second clinical dataset (described below), was applied to the fitted linear mixed-effects model for easier interpretation.

### Mapping logslope to *P*_ROSC_

The mapping from *logslope *to *P*_ROSC _was found from a dataset (mapping-dataset) of pre-shock *logslope *values and corresponding defibrillation outcomes (ROSC or no-ROSC) that has previously been described [20]. The mapping-dataset was collected during cases of out-of-hospital cardiac arrest and ROSC was defined as circulating rhythm for a minimum 10% of the post-shock interval. A marginal logistic regression model for longitudinal data, accounting for correlation between samples from the same patient [21], was fitted to the mapping-dataset using the add-on package 'geepack' to the statistical software R (R Development Core Team, R Foundation for Statistical Computing). The mapping function has the following form:

(2) $$P_{\text{ROSC}}\left(logslope\right)=\frac{e^{\alpha_{0}+\alpha_{1}\cdot logslope}}{1+e^{\alpha_{0}+\alpha_{1}\cdot logslope}}$$

*α*_0 _and *α*_0 _are parameters of the logistic regression estimated during model fitting. The interval-dataset and the mapping-dataset were extracted from the same cardiac arrest episodes. However, since the last 2 seconds of ECG in an interval without chest compressions were not included in the interval-dataset, and we only used the last 2 seconds of ECG before a shock in the mapping-dataset, there is in this respect no overlap of data between the datasets.

### Describing logslope development

Using the statistical software S-plus (Insightful Corporation, Seattle, WA, USA), we fitted a linear mixed-effects model to the interval-dataset [17]. The linear mixed-effects model takes into account the fact that the general level of *logslope *varies between intervals by allowing individual variation in the regression parameters from interval to interval. The *logslope *values are the response variable in the model, and the development of this described with a polynomial in *t*, where *t *is time (seconds) after chest compressions stopped minus 10 seconds (*t *= *t*_org_-10). The subtraction is performed in order to decorrelate the covariates of the model (*t*, *t*^2^, etc). If *i *identifies the interval (cluster), the model for the development of *logslope *with time is given by:

(3) $$logslope_{i}\left(t\right)=\sum_{k=0}^{K}\beta_{k}\cdot t^{k}+\sum_{k=0}^{M}U_{ik}\cdot t^{k}+\varepsilon_{it}$$

*K *is the polynomial degree for the fixed-effects part of the model and *M *the degree for the random-effects part (accounting for variations between intervals) and *K *≥ *M*. Gaussian model residuals *ε*_*it *_are assumed and also a multivariate Gaussian distribution of the random terms *U*_*ik*_, *k *∈ 1, ..., *M*

(4) *f*(*U*_*i*0_, *U*_*i*1_, ..., *U*_*iM*_) = *N*(*0*, Λ)

The S-plus function used to fit the model, lme, has several different possible ways of modelling correlation between residuals within each interval (cluster), and possible heteroschedasticity in the data [17]. We chose the optimal correlation model, variance model (for heteroschedastic data) and the necessary polynomial degree (*K *and *M*), in that order, by comparing Akaike information criterion (AIC) values. Polynomial degrees up to four were tested and among possible models with similar AIC values the model of lowest polynomial degrees was chosen. We did not experiment with other variance covariates than the fitted-model value (software default). For mathematical details about the modelling of correlation between residuals and heteroschedastic data we refer to Pinheiro and Bates [17]. After choosing our final model it was validated by plotting normal probability plots for the model residuals and the random terms, the distribution of residuals against time and fitted-model value and the response against fitted values.

### Calculating *P*_ROSC _development

The linear mixed-effects model was fitted to *logslope *values due to the statistical properties mentioned above, but we wanted to interpret the implications on the *P*_ROSC _scale. More specifically we wanted to find the expected development of *P*_ROSC _for intervals with different starting values. To avoid the random short time variation in *logslope *(and *P*_ROSC_) from influencing the development, we relate each expected development to the starting value of the regression, representing the true underlying behaviour. The results are derived from the fitted linear mixed-effects model (not from specific regression lines for each interval) and the obtained mapping from *logslope *to *P*_ROSC_. Further descriptions of these procedures are given in Appendix 2.

We present the estimated parameters of the chosen linear mixed-effects model and of the logistic regression model for the *logslope *to *P*_ROSC _mapping, along with 95% confidence intervals (CI) or standard deviation (Std) of estimates for all parameters. Further we present estimates of the expected development with time (into the intervals without compressions) of *P*_ROSC _for intervals with different starting values. Approximate 95% CIs for the *P*_ROSC _developments are derived by drawing 1000 simulated parameter values from the model parameters asymptotic distributions, and then recalculating the resulting *P*_ROSC _values for each simulation. The 95% CI for the estimated expected *P*_ROSC _developments are defined as the 95% CI of *P*_ROSC _at a given time into an interval, given a chosen *P*_ROSC _value at the first point of analysis (3 seconds into the intervals).

## Results

**Figure 1:**
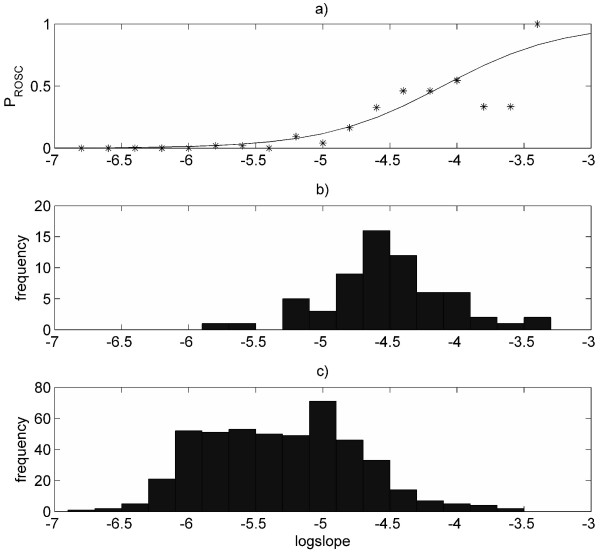
**Logslope to P_ROSC _mapping**. (a) *M*apping (solid line) estimated with logistic regression model. Point-by-point mapping (asterixes) obtained with a standard histogram technique (based on histograms in (b) and (c)). (b) Histogram of pre-shock *logslope *values of defibrillations resulting in return of spontaneous circulation (from mapping-dataset). (c) Histogram of pre-shock *logslope *values of defibrillations resulting in no return of spontaneous circulation (from mapping-dataset).

Data from 530 defibrillation attempts given to 86 patients were included in the mapping-dataset and used to estimate the *logslope *to *P*_ROSC _mapping. In the mapping-dataset *logslope *has an area under the ROSC curve of 0.876 when used to predict which defibrillations will result in ROSC. A histogram of these data is plotted along with the estimated mapping in Figure 1.

*Logslope *values from 911 intervals without chest compressions were included in the interval-dataset and the median interval without chest compressions had five analysis windows. Out of 6229 analysis windows, 17.6% were excluded because of noise in the ECG, leaving 5138. From the complete records of 363 patients, 134 patients had intervals that were included. By comparing AIC values of different candidate models we identified a linear mixed-effects model with an exponential spatial correlation model with nugget effect, polynomial degrees *K *= 1 and *M *= 1, and an exponential variance function. Given that a linear model in *t *(polynomials of degree one) was chosen shifting of the time covariate is not necessary, but in order to simplify calculations a time shift of -3 seconds (*t *= *t*_*org*_-3) was used when estimating the final model.

Estimated model parameters of the logistic regression model for the *logslope *to *P*_ROSC _mapping and of the chosen linear mixed-effects model are given in Table 1. The *logslope *value on average decreases 0.00601 per second in our data range (3 to 27 seconds into an interval without chest compressions). The diagnostic plots confirmed that the linear mixed-effects model is adequate for the data.

**Table 1:** Estimated parameter values of statistical models

**Chosen linear mixed-effects model**		
Parameter	Estimate	95% confidence interval

*β*_0_	-5.224	[-5.263–5.184]
*β*_1_	-0.00601	[-0.00712–0.00490]
Standard deviation (*U*_0_)	0.594	[0.564 0.6324]
Standard deviation (*U*_1_)	0.00708	[0.00564 0.00889]
Correlation (*U*_0_, *U*_1_)	-0.201	[-0.366–0.0416]
Range	3.11	[1.64 5.88]
Nugget	0.520	[0.401 0.629]
Power	-0.598	[-0.805–0.391]

**Logistic regression model**		

Parameter	Estimate	Standard deviation

*α*_0_	9.28	1.51
*α*_1_	2.26	0.315

Estimated model parameters of the chosen linear mixed-effects model and the marginal logistic regression model for the *logslope *to probability of return of spontaneous circulation (*P*_ROSC) _mapping. *β*_0 _and *β*_1 _are the fixed-effects parameters. Standard deviation (*U*_0_), standard deviation (*U*_1_) and correlation (*U*_0_, *U*_0_) give the properties of the zero-mean bivariate Gaussian distribution of the random-effects terms. Range and nugget are parameters in the model for correlation between residuals. Power is the parameter in the variance function. *α*_0 _is the intercept in the logistic regression models linear function, and *α*_1 _represents the change in log-odds ratio (ROSC versus no-ROSC) with a unit change in the covariate (*logslope*).

**Figure 2:**
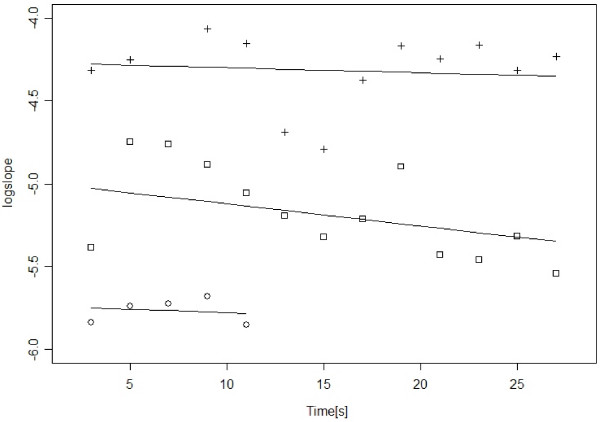
**Calculated *logslope *values and regression lines from the linear mixed-effects model for three intervals without chest compressions**. The linear mixed-effects model is capable of representing that the *logslope *values (plusses, squares and circles) of different intervals are at different levels (have different intercepts) and decrease or increase at different rates (regression lines have different slopes). The fitted model parameters describe how intercepts and slopes are distributed in the dataset.

The data in the interval-dataset and how the linear mixed-effects model represents these are illustrated by Figure 2. The figure shows the *logslope *values and fitted-model values of three intervals. We observe that the intervals are of different length, have *logslope *values at different levels, and that the fitted lines are allowed to have different intercepts and slopes. The fitted values of Standard deviation(*U*_0_), Standard deviation(*U*_1_) and correlation(*U*_0_, *U*_1_) given in Table 1 describe how the intercepts and slopes are distributed in our data.

**Figure 3:**
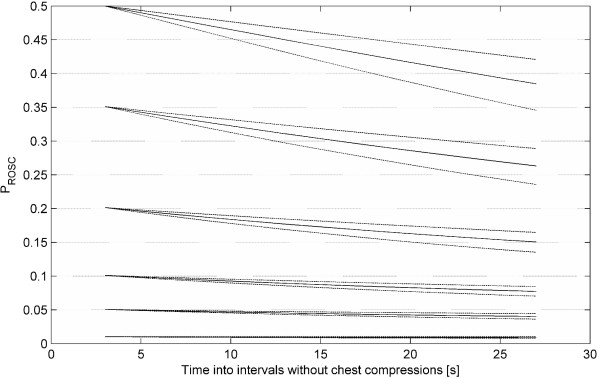
**Estimated developments of mean probability of return of spontaneous circulation in ventricular fibrillation/ventricular tachycardia intervals without chest compression given different starting values of probability of return of spontaneous circulation**. We specified the following starting values (corresponding quantile in our dataset in parenthesis, starting from the top): 0.5(0.97), 0.35(0.92), 0.2(0.80), 0.1(0.6), 0.05(0.38) and 0.01(0.06). The actual starting value of each development deviates slightly from these values because we must integrate out the residual term in our regression model for *logslope *and since we have a non-linear *logslope *to probability of return of spontaneous circulation mapping function. The solid line is the mean probability of return of spontaneous circulation. The dashed lines represent approximate 95% confidence intervals for each of the developments.

Using Equation (8) in Appendix 2 we computed a set of expected developments of *P*_ROSC _given different *P*_ROSC _values 3 seconds into an interval, shown in Figure 3. According to the *β*_0 _parameter, the *logslope *value 3 seconds into an interval has a Gaussian distribution with mean -5.224 and standard deviation 0.594 (Std(*U*_0_)) in our dataset, and we characterise each development by the quantile the initial *logslope *value (corresponding to the chosen initial *P*_ROSC _value) is at in this distribution. About 60% of the intervals have initial *P*_ROSC _below 0.1.

Using the approach described in Appendix 2 we estimate that if all the intervals without chest compressions were shocked with 3 seconds pre-shock pause the mean *P*_ROSC _would be 0.126, while with 27 seconds pause it would be 0.0971. The estimated relative decrease in mean *P*_ROSC_, 1-(0.0971/0.1255) ≈ 0.23, (95% CI: 0.17–0.29) is comparable with that in each of the developments in Figure 3, meaning that independent of the absolute *P*_ROSC _level about 23% of the chance of ROSC will be lost with increasing the pre-shock pause in chest compressions from 3 to 27 seconds.

## Discussion

Our analysis shows that *P*_ROSC_, as determined from ECG analysis, decreases in a steady manner with time in VF/VT intervals without chest compressions for all initial values of *P*_ROSC_. This shows that limiting interruption in chest compressions is important for patients in all states and that every second without perfusion has a negative effect on *P*_ROSC_. The current results therefore give support for a strong focus on improving CPR quality.

Unlike the results of Eftestol et al. [16], the current results show that *P*_ROSC _does not decrease sharply during the first 5 seconds of an interruption. Therefore, a pre-shock pause in compressions of a couple of seconds to ensure the safety of EMS personnel, or to perform rhythm analysis on artefact-free ECG, may be acceptable. Concerning safety, pre-shock pauses of 1 to 2 seconds might be sufficient as it has been argued that the risks of accidental defibrillation of resuscitation providers have been over emphasised [22]. Further, the accuracy of rhythm analysis during ongoing compressions might in the future be improved as a result of either improved artefact removal algorithms or of using dedicated ECG recording electrodes generating fewer artefacts than combined recording and defibrillation electrodes [23]. This will also reduce the need for pre-shock pauses in compressions. Although the current results show that pre-shock interruptions in compressions should be minimised, they do not indicate that it is critical, although favourable, for the outcome of resuscitation to compress during defibrillation. This is because there is no indication of a sharp decrease in *P*_ROSC _during the first few seconds of an interruption.

The effect of pre-shock interruptions in chest compressions on resuscitation outcome or ROSC has also been studied in animals [4-6]. In agreement with the current results all three studies found a clear negative effect of longer pre-shock interruptions. Edelson et al. [7] found strong negative correlation between duration of pre-shock interruptions on first shock success in humans, defined as removal of VF for at least 5 seconds following defibrillation, but no significant effect of pre-shock pause duration on ROSC or survival to hospital discharge. It was stated that one possible reason for this was the limited dataset with lower incidence of ROSC and survival than of shock success with the possibility of a statistical type II error. Eilevstjønn et al. [8] found that the median length of pre-shock interruption of chest compressions for ROSC was 15 seconds versus 18 seconds for no-ROSC shocks (*P *= 0.008), but did not quantify further the negative effect of increasing length of interruptions.

A limitation of our approach is that the effect was studied indirectly by using the established fact that the *P*_ROSC _can be estimated from the ECG waveform [9-12], instead of by direct analysis of the relation between pre-shock pause length and rate of ROSC. The latter could however, only have identified how the mean probability of ROSC for the population would be influenced by increasing pre-shock pauses in CPR, and could not have identified possible differences in the development for cases with different probabilities of ROSC at the start of the pre-shock interval without chest compressions. Using ECG analysis we can estimate *P*_ROSC _continuously for every available interval without chest compressions that follow an interval with chest compressions and, therefore, estimate the expected development of *P*_ROSC _for cases at different starting levels. A further limitation is that we excluded from the analysis all segments where the ECG apparently was affected by a pacemaker and one group of patients was therefore excluded from the analysis. Performing a re-analysis including also the data with noise did however only produce minor changes in the results. At last, the effects of right ventricular dilation and left ventricular contraction occurring during interruptions in compressions, described by Chamberlain et al. [24], may not be reflected in the ECG waveform. The current results may in this respect possibly underestimate the detrimental effect of interrupting compressions.

## Conclusion

We have shown that the probability of ROSC estimated from the ECG decreases in a steady manner with increasing pre-shock pauses in chest compressions. Regardless of initial level, there is a relative decrease in the estimated probability of ROSC of about 23% from 3 to 27 seconds, or, in other words, a 1% relative decrease for every second into such a pause.

## Abbreviations

AIC: Akaike information criterion; CI: confidence interval; CPR: cardiopulmonary resuscitation; ECG: electrocardiogram; *P*_ROSC:_probability of return of spontaneous circulation; ROSC: return of spontaneous circulation; Std: standard deviation; VF: ventricular fibrillation; VT: ventricular tachycardia

## Competing interests

PAS is a member of the board of directors for Laerdal Medical. All other authors declare that they have no competing interests.

## Authors' contributions

KG conceived the study, developed and performed the analysis and drafted the manuscript. JTK contributed to the development of the analysis methods. JKJ and PAS contributed to the acquisition and preparation of data. TE contributed to the conception of the study and to the preparation of data. All authors have revised and approved the final manuscript.

## Appendix 1

**Figure 4:**
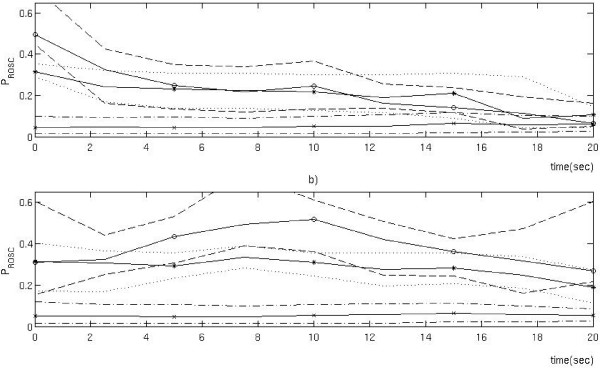
**Two plots illustrating the problem with the analysis performed in the article by Eftestol et al**. (a) The original plot from Eftestol et al.'s [16] article where each case was assigned to one of three subgroups ('high', 'medium' and 'low') according to their initial value of probability of return of spontaneous circulation (*P*_ROSC_). The time axis refers to time into intervals without compressions. The median *P*_ROSC _in each group is represented with a solid line and the various broken lines represent the 25th and 75th percentiles for each group. The median of the 'high' group differs from the median of the 'medium' group only for the first 5 seconds, and the median of the 'low' group appears to be increasing towards the end. (b) This plot was generated with the same analysis as (a), but here the cases were assigned to the three subgroups according to their *P*_ROSC _value at 10 seconds. From the difference between plots (a) and (b), it is evident that the short time random variations in the *P*_ROSC _estimates greatly affect the results of this form of analysis.

This section describes why we claim that the analysis in Eftestol et al. [16] was flawed. That particular paper is the only previous study to use ECG analysis on clinical data to investigate the effects of interrupting chest compressions on the probability of ROSC. In Eftestol et al. [16], the ECG segments (from pre-shock VF intervals without chest compressions) were divided into three groups, one for those with an initially high *P*_ROSC _value (the first *P*_ROSC _value in the segment >0.4), one with medium initial values (0.25< first *P*_ROSC _< 0.40), and one with low initial values (<0.25). After this the development with time of the median of *P*_ROSC _in the segments in these three groups was calculated. However, there are quite large random short time variations, or measurement noise, in the *P*_ROSC _estimate used [25], and these influence the group to which the segments are assigned in the first place. Therefore, for the group of segments with high initial *P*_ROSC _value a large proportion of the segments will have high values only at the start of the segment. The median of each group will approach the median of all the segments for the consecutive measurements. We can demonstrate this by re-analysis of the original data from Eftestol et al. [16], but assign the segments to three groups according to the *P*_ROSC _values at 10 seconds into the segments rather than according to the initial values. The result of this is shown together with the original plot from Eftestol et al. [16] in Figure 4. If the *P*_ROSC _estimate from each ECG segment had little or no random variation between consecutive measurements, this modification of the analysis should have little effect on the results. However, changing the time at which the assignment to groups is performed totally changes the plot and we must, therefore, conclude that the analysis is flawed. The inadequate analysis led to unjustified conclusions, most prominently for the 'high' and 'low' subgroups. For cases with a high initial level a dramatic decrease in median *P*_ROSC _(from 0.5 to 0.25) with only 5 seconds interruption in chest compressions was falsely indicated, and for the group of cases with low initial *P*_ROSC_, representing 85% of the total number of cases, it was indicated that the median *P*_ROSC _is stable or slightly increasing during interruptions of up to 20 seconds, despite there being no coronary perfusion. It should be noted that two of the authors (TE and PAS) of the criticised article are also co-authors of the current article.

## Appendix 2

If we choose value *x *at *t *= 0 for the regression to *logslope*, for a hypothetical interval *h *by choosing *U*_*h*0 _= *x *- *β*_0_, we can estimate the development of the mean *P*_ROSC _for intervals with a specific starting value. In this way the estimated development is not influenced by the random short time variation in *logslope *at *t *= 0, represented by *ε*_*h*0_. By analogy, this was the problem with the analysis performed by Eftestol et al. [16]. With the above choice of *U*_*h*0 _the marginal distribution of the other random terms is given by:

(5) $$f_{m}\left(U_{h1},...,U_{hM}\right)=\frac{f\left(x-\beta_{0},U_{h1},...,U_{hM}\right)}{\int_{-\infty }^{\infty }..\int_{-\infty }^{\infty }f\left(x-\beta_{0},U_{h1},...,U_{hM}\right)dU_{h1},....dU_{hM}}$$

the model for the *logslope *at *t *by:

(6) $$logslope_{h}\left(t,U_{h1},...,U_{hM}|U_{h0}+\beta_{0}=x\right)=x+\sum_{k=1}^{K}\beta_{k}\cdot t^{k}+\sum_{k=1}^{M}U_{hk}\cdot t^{k}+\varepsilon_{ht}$$

and, using Equation (2) for the *logslope *to *P*_ROSC _mapping, the model for *P*_ROSC _at *t *is given by:

(7) $$P_{ROSC,h}\left(t,U_{h1},...,U_{hM}|U_{h0}+\beta_{0}=x\right)=\frac{\exp\left(\alpha_{0}+\alpha_{1}\cdot \left(x+\sum_{k=1}^{K}\beta_{k}\cdot t^{k}+\sum_{k=1}^{M}U_{hk}\cdot t^{k}+\varepsilon_{ht}\right)\right)}{1+\exp\left(\alpha_{0}+\alpha_{1}\cdot \left(x+\sum_{k=1}^{K}\beta_{k}\cdot t^{k}+\sum_{k=1}^{M}U_{hk}\cdot t^{k}+\varepsilon_{ht}\right)\right)}$$

Then the expected *P*_ROSC _development given the chosen initial value can be calculated by:

(8) $$\begin{array}{l} E\left[P_{ROSC,h}\left(t\right)|U_{h0}+\beta_{0}=x\right]=\\ \int_{-\infty }^{\infty }..\int_{-\infty }^{\infty }P_{ROSC,h}\left(t,U_{h1},...,U_{hM}|U_{h0}+\beta_{0}=x\right)\cdot f_{m}\left(U_{h1},...,U_{hM}\right)\cdot f\left(\varepsilon_{ht}\right)dU_{h1},..,dU_{hM},d\varepsilon_{ht} \end{array}$$

By choosing different values for *x *we can calculate the expected *P*_ROSC _development for cases with different starting values. For a given starting value of *P*_ROSC_, the corresponding *logslope *value *x *is found using Equation (2). If we make no specific choice of *logslope *at *t *= 0, but modify Equation (8) to integrate out the random intercept U_h0 _as well as the other random terms, we can obtain an estimate for how the mean *P*_ROSC _develops with time, pooling together cases at all *P*_ROSC _levels.

## Pre-publication history

The pre-publication history for this paper can be accessed here:


